# Effect and safety of Huangqi-Guizhi-Wuwu Decoction and Erxian Decoction in the treatment of frozen shoulder: A protocol for systematic review and meta-analysis

**DOI:** 10.1097/MD.0000000000020540

**Published:** 2020-06-05

**Authors:** You-Wei Zhang, Chao Jiang, Xiao-Hong Li, Kai Li

**Affiliations:** a Department of Orthopedics, Baoji Central Hospital, Baoji, Shaanxi; b The Third Department of Neurology, The Second Affiliated Hospital of Xi’an Medical University, Xi’an; c Department of Emergency, Longhua Hospital Shanghai University of Traditional Chinese Medicine, Shanghai, PR China.

**Keywords:** effect, Erxian Decoction, frozen shoulder, Huangqi-Guizhi-Wuwu Decoction, randomized controlled trial, safety

## Abstract

**Background:**

The purpose of this study is to evaluate the effect and safety of Huangqi-Guizhi-Wuwu Decoction (HGWD) and Erxian Decoction (EXD) in the treatment of frozen shoulder (FS).

**Methods:**

We will compressively search potential randomized controlled trials from electronic databases of MEDLINE, EMBASE, Cochrane Library, CINAHL, PsycINFO, Web of Science, Allied and Complementary Medicine Database, Google Scholar, and China National Knowledge Infrastructure. We will search all of them from inception of each electronic database up to the present without language limitations. Two researchers will conduct selection of study, data extraction, and study quality evaluation independently. Study quality will be identified using Cochrane risk of bias tool. Statistical analysis will be performed using RevMan 5.3 software.

**Results:**

This study will summarize high quality evidence of randomized controlled trials on exploring the effect and safety of HGWD and EXD in the treatment of FS.

**Conclusions:**

The results of this study will provide helpful evidence of the effect and safety of HGWD and EXD in the treatment of FS to facilitate the clinical practice and guideline development.

**Study registration number:**

INPLASY202040070.

## 1 Introduction

Frozen shoulder (FS), also known as adhesive capsulitis, is a very common shoulder disease, which is characterized by progressive shoulder pain and restriction of shoulder motion.^[1–6]^ It is estimated that its incidence is 3% to 5% in the general population,^[7]^ and this number is up to 20% in patients with diabetes.^[8]^ Although a wide range of managements are available for FS, their efficacy is still limited.^[9–17]^ Recent studies have reported that Huangqi-Guizhi-Wuwu Decoction (HGWD) and Erxian Decoction (EXD) effectively treat patients with FS.^[18–21]^ However, presently, no published study has systematically assessed the effect and safety of HGWD and EXD for the treatment of FS. Thus, this study will investigate the effect and safety of HGWD and EXD for patients with FS.

## 2 Methods

### 2.1 Study registration

This study has registered on INPLASY202040070. We have reported this study according to the guidelines of preferred reporting items for systematic review and meta-analysis protocols statement.^[22]^

### 2.2 Eligibility criteria

#### 2.2.1 Types of studies

Only randomized controlled trials (RCTs) of HGWD and EXD in the treatment of FS will be included. Language and publication status is not limited. Any other studies will be excluded, such as case reports, case series, reviews, non-RCTs, and quasi-RCTs.

#### 2.2.2 Types of interventions

##### 2.2.2.1 Experimental interventions

The treatment group will use HGWD and EXD with no limitation of dosage, frequency and treatment period.

##### 2.2.2.2 Control interventions

As for the comparators, they could be any treatments, such as placebo, western medicine. However, patients who received HGWD or EXD or combination of HGWD and EXD, the trials will be rejected.

#### 2.2.3 Types of patients

All adult participants (18 years old or above) who were diagnosed as FZ will be considered for inclusion, regardless country, race, and gender.

#### 2.2.4 Types of outcome measurements

Primary outcome is shoulder pain intensity, as measured by any validated pain scales.

Secondary outcomes are shoulder function, as checked by any validated disability Indexes; quality of life, as evaluated by any validated related scores; and any expected or unexpected adverse events.

### 2.3 Search strategy

We will compressively retrieve the following electronic databases of MEDLINE, EMBASE, Cochrane Library, CINAHL, PsycINFO, Web of Science, Allied and Complementary Medicine Database, Google Scholar, and China National Knowledge Infrastructure. We will collect all electronic database sources from inception of each electronic database up to the present without language and publication status limitations. The example of search strategy with details of MEDLINE is built (Table 1). The similar search strategies will be utilized to other electronic databases.

**Table 1:**
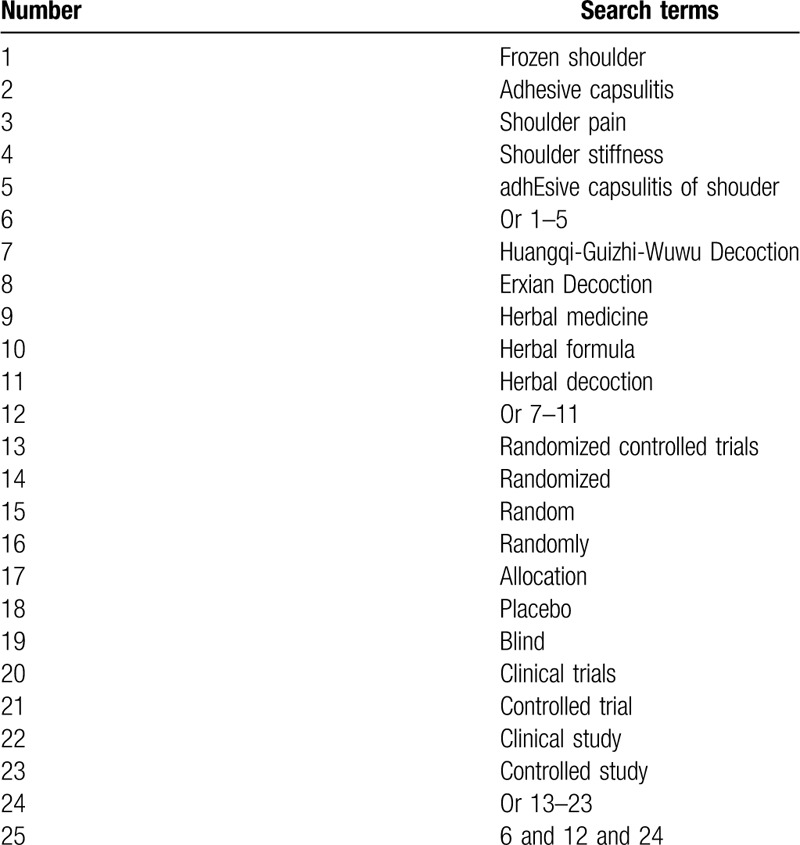
Search strategy sample of MEDLINE.

Additionally, we will also search Google scholar, conference proceedings, clinical registration websites, and reference lists of associated reviews.

### 2.4 Data collection and analysis

#### 2.4.1 Study selection

All searched records will be imported into Endnote X7 and all duplicated studies will be removed by 2 independent qualified evaluators. The titles and abstracts of all selected studies will be screened or scanned based on the predefined eligibility criteria. All irrelevant studies will be excluded. The remaining studies will be checked by reading full texts. Any different opinions of study selection between 2 evaluators will be solved by another experienced evaluator via discussion or consultation. The whole procedures of study selection will be shown in a flowchart with specific reasons for all removed trials.

#### 2.4.2 Data extraction

Two qualified evaluators will independently extract data from the selected trials. Any different views between 2 evaluators will be solved by a third evaluator through discussion. The extracted date mainly includes general study and patient information, such as country, year of publication, first author; study setting; study design; sample size; interventions and controls; outcomes; adverse events; and any other essential data.

#### 2.4.3 Dealing with missing data

If missing or unclear data occurs, we will contact primary authors to obtain it. In the absence of reply, only data at hand will be analyzed and will discuss its potential affects to the conclusions.

### 2.5 Study quality assessment

Two evaluators will independently evaluate study quality for each eligible trial utilizing Cochrane risk of bias tool. This tool covers 7 aspects, and each one is graded as high, unclear and low risk of bias. Any divergences will be worked out by a third evaluator through discussion.

### 2.6 Subgroup analysis

Subgroup analysis will be addressed to check the potential heterogeneity and inconsistency based on the different study or participant characteristics, treatments, controls, and outcome measurements.

### 2.7 Sensitivity analysis

Sensitivity analysis will be investigated to identify the robustness and stability of study results by removing low quality trials.

### 2.8 Reporting bias

Reporting bias will be checked using funnel plot and Egger’ regression test if sufficient eligible trials (over 10 RCTs) are included.^[23,24]^

### 2.9 Quality of evidence

The quality of evidence for each outcome will be assessed through a guideline development tool (GRADEpro GDT, https://gradepro.org/). Two evaluators will independently evaluate it. Any different opinions will be solved by a third experienced evaluator through discussion.

### 2.10 Data synthesis

In this study, RevMan 5.3 software will be performed to analyze outcome data and to carry out meta-analysis. All continuous data are calculated as mean difference or standardized mean difference and 95% confidence intervals (CIs), while all dichotomous data will be presented as risk ratio and 95% CIs. The *I*^*2*^ statistics will be used to check statistical heterogeneity across included trials. A value of *I*^*2*^ ≤50% is considered as having homogeneous and data will be pooled using a fixed-effect model. If over two eligible trials are included, we will conduct a meta-analysis. On the other hand, a value of *I*^*2*^ >50% indicates significant heterogeneity, and data will be synthesized using a random-effect model. We will investigate the reasons for the existence of substantial heterogeneity from several aspects, such as characteristics of study and patient, and interventions. The sources of heterogeneity will be further examined using sensitivity analysis.

## 3 Discussion

Currently, HGWD and EXD have been utilized for the treatment of FS. Several published studies have explored the comparative effect and safety of HGWD and EXD for the treatment of FS. However, so far, no systematic review and meta-analysis has been performed to appraise the comparative efficacy and acceptability of HGWD and EXD for the treatment of FS. Therefore, it is very important to assess the effect and safety of HGWD and EXD for FS.

To our best knowledge, the present study firstly investigates the effect and safety of HGWD and EXD for FS. The findings of this study help to determine whether HGWD and EXD are effective and safety for FS management. It may provide evidence for both guideline recommendations of clinical practice and further studies.

## 4 Ethics and dissemination

Ethical approval is not required as this study is a review of previously published studies. We expect to publish the results of this study on a peer-reviewed journal.

## Author contributions

**Conceptualization:** You-Wei Zhang, Chao Jiang, Xiao-Hong Li, Kai Li.

**Data curation:** Chao Jiang.

**Formal analysis:** You-Wei Zhang, Chao Jiang, Xiao-Hong Li.

**Funding acquisition:** Kai Li.

**Investigation:** Kai Li.

**Methodology:** You-Wei Zhang, Chao Jiang.

**Project administration:** Kai Li.

**Resources:** You-Wei Zhang, Chao Jiang, Xiao-Hong Li.

**Software:** You-Wei Zhang, Chao Jiang, Xiao-Hong Li.

**Supervision:** Kai Li.

**Validation:** You-Wei Zhang, Chao Jiang, Kai Li.

**Visualization:** Chao Jiang, Xiao-Hong Li, Kai Li.

**Writing – original draft:** You-Wei Zhang, Chao Jiang, Xiao-Hong Li, Kai Li.

**Writing – review & editing:** You-Wei Zhang, Chao Jiang, Xiao-Hong Li, Kai Li.
